# Service Evaluation of Diagnostic Evolution in Psychiatric Patients at Benazir Bhutto Hospital: Comparing OPD and ER Admissions

**DOI:** 10.1192/bjo.2024.476

**Published:** 2024-08-01

**Authors:** Naima Gul, Asad Nizami

**Affiliations:** Institute of Psychiatry, Rawalpindi Medical University, Rawalpindi, Pakistan

## Abstract

**Aims:**

This project evaluated the accuracy and evolution of psychiatric diagnoses in patients admitted through the Outpatient Department (OPD) and Emergency Room (ER) at Benazir Bhutto Hospital. It aimed to understand the factors contributing to diagnostic changes, especially the impact of comorbid conditions and interdisciplinary discussions.

**Methods:**

Over an eight-month period, this study reviewed 200 patient records from the psychiatric department. It compared initial psychiatric diagnoses from OPD and ER admissions with final diagnoses at discharge. The evaluation examined the influence of ward round discussions, serial mental state examinations, and newly identified comorbid medical conditions, such as thyroid disorders and neurological issues, on diagnostic changes.

**Results:**

Analysis showed that 38.2% of ER admissions had a revised diagnosis by discharge, compared with 22.5% from OPD. Initial diagnoses primarily included major depressive disorder (30.1%) and bipolar disorder (27.2%). By discharge, increases were observed in personality disorders (up by 18.3%) and substance use disorders (up by 14.7%). Comorbid medical conditions were newly diagnosed in 26.8% of patients. Factors influencing diagnostic changes included ward round discussions (57.3%), serial mental state examinations (40.2%), lab findings (33.5%), and medical/interdisciplinary consultations (29.6%).

**Conclusion:**

The service evaluation at Benazir Bhutto Hospital reveals significant diagnostic evolution in psychiatric care, more pronounced in ER admissions. The identification of additional disorders and comorbid medical conditions highlights the necessity for comprehensive, ongoing psychiatric assessment. Lab findings and interdisciplinary consultations played a crucial role in refining diagnoses, suggesting the importance of an integrated care approach. Recommendations include improving initial diagnostic processes in ER settings and strengthening interdisciplinary communication to enhance accuracy in psychiatric diagnosis and patient treatment outcomes.

